# Supplementary material for: A community-based vector control intervention “Slash and Clear” implemented in two onchocerciasis-endemic foci in South Sudan
Source: PLoS Negl Trop Dis. 2025 Jul 23;19(7):e0013309. doi: 10.1371/journal.pntd.0013309 (PMC12306731; doi:10.1371/journal.pntd.0013309)
Supplement: S1 Table — (DOCX) [file pntd.0013309.s001.docx]

| **River** | **Intervention** | **Control(s)** | **MBRs Geometric Mean difference between intervention and Control** | **d.f.** | **F** | **t** | **p-value**  **(2-sided)** | **95% CI** | |
| --- | --- | --- | --- | --- | --- | --- | --- | --- | --- |
| Naam | Dogoyabolu | Main Bridge | -68.78 | 26 | 1.10 | -0.203 | 0.84 | **Lower** | **Upper** |
|  |  |  |  |  |  |  |  | -2.03 | 3.59 |
|  | Dogoyabolu | Domalira | 89.80 | 26 | 0.39 | 0.411 | 0.68 | -2.05 | 3.07 |
|  | Dogoyabolu | Main Bridge + Domalira | 25.75 | 40 | 1.08 | 0.108 | 0.91 | -2.15 | 2.40 |

**S1 Table. Results of un-paired t-test statistic between intervention and control sites along Naam River in Mvolo County, Western Equatoria, South Sudan**
