# Supplementary material for: A community-based vector control intervention “Slash and Clear” implemented in two onchocerciasis-endemic foci in South Sudan
Source: PLoS Negl Trop Dis. 2025 Jul 23;19(7):e0013309. doi: 10.1371/journal.pntd.0013309 (PMC12306731; doi:10.1371/journal.pntd.0013309)
Supplement: S2 Table — (DOCX) [file pntd.0013309.s002.docx]

**S2 Table. Results of un-paired t-test statistic between intervention and control sites along Yei River in Mundri West County Western Equatoria, South Sudan.**

| **River** | **Intervention** | **Control(s)** | **MBRs Mean difference between intervention and Control** | **d.f.** | **F** | **t** | **p-value (2-sided)** | **95% CI** | |
| --- | --- | --- | --- | --- | --- | --- | --- | --- | --- |
| Yei | Wulikori | Dongoro | 480.50 | 14 | 0.03 | 0.678 | 0.51 | Lower | Upper |
|  |  |  |  |  |  |  |  | -1,039.74 | 2,000.74 |
|  | Tawa | Boro | 393.50 | 14 | 0.00 | 0.674 | 0.51 | -578.82 | 1,109.13 |
|  | Wulikori + Tawa | Dongoro +Boro | 372.83 | 22 | 0.016 | 0.864 | 0.39 | -508.58 | 1,254.24 |
|  |  |  |  |  |  |  |  |  |  |

_MBRs=Monthly Biting Rtaes; d.f.=degree of freedom; CI=Confidence Interval_
