# Supplementary material for: A community-based vector control intervention “Slash and Clear” implemented in two onchocerciasis-endemic foci in South Sudan
Source: PLoS Negl Trop Dis. 2025 Jul 23;19(7):e0013309. doi: 10.1371/journal.pntd.0013309 (PMC12306731; doi:10.1371/journal.pntd.0013309)
Supplement: Attachment — Submitted filename: REPORT PLOS NEGLECETD TROPICAL DISEASES_NWANE.docx [file pntd.0013309.s003.docx]

**REPORT PLOS NEGLECETD TROPICAL DISEASES**

Manuscript Number: PNTD-D-25-00522

**A community-based vector control intervention “Slash and Clear” implemented in two onchocerciasis-endemic foci in South Sudan**

**Summary**

This paper, whose data present a trial of physical control of onchocerciasis vectors, is very important. These data allow us to further explore the contours associated with the implementation of this black fly control strategy to eliminate onchocerciasis in endemic areas. However, the data presented in this paper suggest that a single round of S&C cannot impact MBR in the medium or long-term. The success of this strategy requires repeated rounds of S&C implementation each year with the right timing to sustainably decrease black fly biting rates.

This paper can be published the following raised comments and suggestions are considered.

**Line 36**: Separate Simuliumdamnosum; “Senso lato” replace by “sensu lato”

**Line 39**: Write Tau

**Line 96**: The authors should remove one Sudan in “Sudan Sudan”

**Line 102**: No abbreviation Write River not R. and replace “river” by watercourse

**Lines 110-111**: Give the names of the 2 control sites ands give the real distance between intervention and control sites. This helps in understanding the provided results

**Lines 131-132**: Please revise this sentence as follows “Populations practice subsistence agriculture based on the cultivation of cassava .........”

**Lines 168-169**: The authors must specify the number of blackfly collection points in each section of the Naam and Yei rivers.

**Line 182**: Amadi is not presented in the section describing the Mvolo or Mundri Counties

**Line 192**: The authors previously said in the section of method that S&C took 2 days and was conducted by six youths; Please can you clarify this?

The number of young people who conducted S&C in this intervention site must be specified; this must be clear for the 2 intervention sites

**Line 189-190**: S&C performed 700 m upstream and 200 m downstream of the intervention sites. Does this mean that the section between upstream and downstream was not affected by this intervention?

**Lines 240 et 244**: The authors should present the number of larvae collected per area of substrate

What is the meaning of ++++ in Table 1? The term “larvae and pupae” appears several times in the legend, and the authors need a clear way of presenting it.

**Lines 253-255**: The authors should move these sentences in the section of methods

**Line 256**: After giving the unit of the monthly biting rate or daily biting rate on the method section (bite per person per month or bite per person per day), use their abbreviations bi/p/m or bi/p/d in the section of results

**Line 260** : Write per day

In table 2, Daily Biting Rate (DBR); replace DBR by (bi/p/d)

**Lines 269-270**: The authors must move this sentence in the section of discussion

**Lines 276-280**: That doesn't seem to convince me. There are already 2 control sites and one intervention site shown in the figure. Which of these are affected by the reduction you refer to in the 4 months following S&C? It seems rather contrary to me to take a good look at the figure and propose an appropriate comment.

On **line 192**, the authors say that S&C was implemented in March and on figure 6 the arrow is pointing to April. Can you clarify this misunderstanding? My comment about reduction rate of biting in Naam, comes back here

Table 2 Write “degrees”

**Lines: 335-338**: What makes you conclude that it's S. damnosum when you're talking about limited data? What makes you conclude that it's S. damnosum when you're talking about limited data?

**Line 371**: In this part of the discussion, the authors should mention the contamination of the intervention sites by the control sites, which are all nearby, as black flies can move over long distances

**Line 440-441**: The authors should revise the sentence
