# Supplementary material for: A community-based vector control intervention “Slash and Clear” implemented in two onchocerciasis-endemic foci in South Sudan
Source: PLoS Negl Trop Dis. 2025 Jul 23;19(7):e0013309. doi: 10.1371/journal.pntd.0013309 (PMC12306731; doi:10.1371/journal.pntd.0013309)
Supplement: Attachment — Submitted filename: Feedback_S_C PloS manuscript.docx [file pntd.0013309.s004.docx]

A community-based vector control intervention “Slash and Clear” implemented in two

onchocerciasis-endemic foci in South Sudan

PNTD-D-25-00522

**Overall feedback**

This is a great work especially as slash and clear has not been overly exploited as a vector control tool. However, I had a bit of concern especially with the study design. It was unclear to me if this was a case control study or an intervention study (before-after).

Secondly, in the abstract, I think the results and conclusion do not emphasize the purpose of the work. From my understand, the study was to assess the impact of the community-based vector control S&C. The other results such as the BR being higher in one season than the other, etc., are more secondary outcomes but they overshadowed the rationale behind the study. I think the main objective should stand out esp. in the abstract. This was however elaborated in the author summary.

Line review:

Line 96: Double Sudan. Delete one

Line 71, 149, 153, etc.: Blackfly or Black fly? Could you select one and be consistent with that.

Line 159-161: Add that larval density at the breeding sites was determined, as you have indicated the infestation levels in the results (line 240; 243; 244…)

Line168-169: How many collection points for each river? Will be good to mention.

Line 191-192: Was the S&C for the 7 days done consecutively? If so, kindly indicate.

Line 302: Add GAM, which is then used in line 308.

Line 308: The GAM model…, can you take out the “model”?
