# Supplementary material for: A community-based vector control intervention “Slash and Clear” implemented in two onchocerciasis-endemic foci in South Sudan
Source: PLoS Negl Trop Dis. 2025 Jul 23;19(7):e0013309. doi: 10.1371/journal.pntd.0013309 (PMC12306731; doi:10.1371/journal.pntd.0013309)
Supplement: Attachment — Submitted filename: 28.05.25Response to reviewersFinal.docx [file pntd.0013309.s007.docx]

**Response to reviewers**

**Reviewer #1:** This is an important paper in the context of the potential approaches to controlling blackflies using the Slash and Clear (S&C) method by reducing the vegetation substrate in blackfly breeding sites which has been used in other settings. I feel that the authors need to emphasise the rationale that vector control is an important adjunct to the overall objective of elimination of onchocerciasis in Africa in combination with ivermectin MDA. The effectiveness of vector control (using insecticide) in certain settings needs to be expressed (eg Bioko, Uganda foci) assuming they are isolated which the sites studied in South Sudan are not. However, the study does show that the challenges of this approach really cannot be used and/or sustained over the vast areas of oncho endemicity placing in doubt the likelihood that S&C can be used at the necessary scale. This needs to be contextualised as the feasibility of insecticide is also a major challenge for environmental, cost and sustainability reasons. I would encourage the authors to add a paragraph raising these issues. Essentially, if WHO targets for oncho elimination are to be reached on the scale required in the diverse oncho geographies then vector control, however desirable, is not likely to have a meaningful role.

**Response**

**We now added the following paragraph in the introduction**

**“Since the 1990s, global efforts to control and eliminate onchocerciasis have relied on large-scale preventive chemotherapy through mass drug administration (MDA) of ivermectin, which kills the larval stages (microfilariae) [1]. In some areas, MDA has been complemented by vector control strategies, such as aerial larviciding, to reduce blackfly populations [1]. Together, these approaches form the basis of onchocerciasis control and elimination programmes. Vector control is a critical adjunct to ivermectin MDA for achieving onchocerciasis elimination, particularly in settings where: vector densities are high, transmission persists despite MDA, and especially in geographically isolated foci where it has proven highly effective, such as in in the Victoria Nile [5] and Mount Elgon [6] foci in Uganda and Bioko Island in Equatorial Guinea [7]. However, in South Sudan where are onchocerciasis foci are not geographically isolated and rivers may contain continuous breeding sites and where resources are limited, implementing vector control is challenging.”**

**We also added the following references:**

**Katabarwa MN, Habomugisha P, et al. "Historical Elimination of Onchocerciasis from Victoria Nile Focus in Central Uganda Verified Using WHO Criteria." The American Journal of Tropical Medicine and Hygiene, 2020.**

**Katabarwa MN, Lakwo TL, Habomugisha P, et al. "Transmission of Onchocerca volvulus by Simulium neavei in Mount Elgon Focus of Eastern Uganda." The American Journal of Tropical Medicine and Hygiene, 2014.**

**Traoré S, Wilson MD, Sima A, et al. "The elimination of the onchocerciasis vector from the island of Bioko as a result of larviciding by the WHO African Programme for Onchocerciasis Control." Acta Tropica, 2009.**

**Reviewer #2**

Table 2. The heading for the last column should be Daily Mean Biting Rate (DBR).

**Response**

**We now changed the heading to Mean Daily Biting Rate (DBR).**

**Reviewer #2**

• Figure 3. Does the figure show clearly an evening rise in biting rates at Tawa (Lines 268 – 269)? This is not clear from the figure.

**Response**

**We now state in the results “At the Dongoro and Tawa sites, morning peaks were not clearly observed and in Tawa there was no increase of biting rates in the evening.”**

**Reviewer #2**

• Lines 305 – 307. How confident are the authors about the reliability of the log transformed data on mean biting rates on the Naam River?

**Response**

**The log transformed data on biting rates along R. Naam is quite reliable since it is a standard method in medical statistics (Kirkwood and Sterne, 2003). Monitoring data for the biting rates along R. Naam contained some zeros in some months making it skewed. Therefore, there was need to transform MBRs to a new scale using logarithm to base e (or natural logarithm) before performing any analysis. Prior to transformation, a constant value of 0.001 was added to MBRs across sites because some of them contained zeros. Logarithm transformation was performed in Microsoft Excel software before exporting them to SPSS version 26.0 (IBM, USA) for analysis.**

**Reviewer #2:**

Line.111. What was the approximate distance between the study and control sites?

**Response**

**The distances between intervention and control sites ranged from 1.5-2.5 km.** **The short distances between intervention and control sites were attributed to the short stretch of *S. damnosum* breeding along River Naam in Mvolo. This has been stated in the text.**

**Reviewer #2**

The figures are not titled and are rather blurred, but in Figure 1, it looks as if the study site was flanked by the control sites; if this was the case can the authors comment on whether the interventions at the study site could have contaminated the findings at the control sites?

**Response**

**The title of the Figures are in the text.**

**Indeed interventions at the study site could have contaminated the findings at the control sites.**

**We state “The contamination of intervention sites by control sites in this study was attributed to the short distances between control and intervention sites. In Uganda, a country neighbouring South Sudan, *S. damnosum* s.l was reported to disperse up to a distance of 55 km from the breeding site [20].”**

**Reviewer #2**

• Lines 150-151: The sentence “Prospections in the two rivers (Naam and Yei) were conducted by experienced technical staff,…” could be reworded for clarity. The word “prospected” usually applies to the search for mineral deposits. For example: The rivers Naam and Yei were surveyed by experienced technical staff,…”. Lines 153, 180, 238, 415, Table 1: “prospections” could be replaced by “surveys”.

**Response**

**Prospections is a term used in WHO documents for entomological surveys**

**However we agree to now use the word surveyed and surveys**

**Reviewer #2**

• Lines 240-241: can the authors give a figure for the number of remaining sites along the River Naam? Table 1 suggests there were four.

**Response**

**Four sites were identified but not the entire river was explored.**

**Reviewer #2**

I am concerned that the ethical issues around human landing catches are not articulated and there is no reference to this aspect of the study. The oncho community are seeking to find alternative approaches to the issue and this needs to be referred to

**Response**

**We now state in the methods**

**“To measure human-biting rates of blackflies the human landing catches (HLC) method was chosen, which is still considered by WHO as the gold standard method for this purpose, Esperanza Window traps are an alternative to collect blackflies. While Esperanza traps offer ethical and operational advantages by reducing human exposure to blackfly bites, they currently face technical limitations: They measure attraction rather than true biting rates, show variable correlation with HLC data, and are not yet validated as a reliable substitute for entomological assessments required for onchocerciasis elimination verification.”**

**We also added that the volunteers were treated with ivermectin.**

**We now mention this in the discussion.**

**Reviewer #2:**

Lines 343, 424. Can the authors explain what is meant by emboldened rocks?

**Response**

**We now use the term “prominent rocks”**

**Reviewer #2**

• Lines 359-361. It is not clear from this sentence whether the Tawa and Boro sites are located in savannah regions. Could the authors clarify?

**Response**

**We now clarify “Similar but less pronounced morning biting peaks were also observed in Tawa and Boro, two savannah sites along the River Yei. These patterns are in line with earlier reports from all sites along the River Naam [8].”**

**Reviewer #2**

• Lines 366 – 368. Can the authors provide evidence in support of the negative effect of high temperatures on black fly breeding behaviour, even in the more stable forested areas?

**Response**

**We were referring to the effect of the high temperatures on the blackfly biting behaviour and not breeding behaviour. We have now made a revision in the text to read: “It is known that high temperature and low relative humidity usually affect diurnal biting patterns. This explains why there are unimodal and bimodal biting patterns. In the savannah, there are usually very high temperatures and low relative humidity because the terrain is dominated by grassland and spotted trees. Most of the rivers flow in the open compared to those in the forest where the canopies contribute in lowering the temperature. This therefore explains why the forest ecological habitats have moderate temperatures and relative humidity, thus sustaining a stable blackfly biting pattern”.**

**Reviewer #2**

The paper could be streamlined in terms of language and punctuation, for better flow of the narrative. It would have been helpful for a native English speaker to have reviewed the text before submission.

• Lines 84, 196, 451, 460: AMREF Health Africa should be Amref Health Africa. Amref is no longer an acronym after the name change of the organization.

• It is quite hard to match the figures to the text since none of the figures are titled. In addition, they appear to be rather blurred.

**Response**

**The paper has been reviewed and edited by a native English speaker**

**We now always use Amref Health Africa**

**All figures are titled in the text**

**Reviewer #3**

**Summary**

This paper, whose data present a trial of physical control of onchocerciasis vectors, is very important. These data allow us to further explore the contours associated with the implementation of this black fly control strategy to eliminate onchocerciasis in endemic areas. However, the data presented in this paper suggest that a single round of S&C cannot impact MBR in the medium or long-term. The success of this strategy requires repeated rounds of S&C implementation each year with the right timing to sustainably decrease black fly biting rates.

**Response**

**We agree with this summary**

**Reviewer #3**

This paper can be published the following raised comments and suggestions are considered.

**Line 36**: Separate Simuliumdamnosum; “Senso lato” replace by “sensu lato”

**Line 39**: Write Tau

**Line 96**: The authors should remove one Sudan in “Sudan Sudan”

**Line 102**: No abbreviation Write River not R. and replace “river” by watercourse

**Response**

**All errors were corrected**

**Reviewer #3**

**Lines 110-111**: Give the names of the 2 control sites ands give the real distance between intervention and control sites. This helps in understanding the provided results

**Response**

**We now state “The Domilara and Main Bridge control sites were located 1.5 km and 2.5 km, respectively, from the intervention site. The short distances between the intervention and control sites were due to the limited stretch of *Simulium damnosum* breeding habitat along the River Naam in Mvolo****.”**

**Reviewer #3**

**Lines 131-132**: Please revise this sentence as follows “Populations practice subsistence agriculture based on the cultivation of cassava .........”

**Response**

**Sentence was revised as proposed**

**Reviewer #3**

**Lines 168-169**: The authors must specify the number of blackfly collection points in each section of the Naam and Yei rivers.

**Response**

**Blackfly collections were conducted at three catching points along R. Naam and four catching points along the Yei River.**

**Reviewer #3**

**Line 182**: Amadi is not presented in the section describing the Mvolo or Mundri Counties

**Response**

**A brief description of Amadi has been provided under Mundri West. We now state "Amadi Payam is an administrative division within Mundri West County, predominantly inhabited by the Moru and Avukaya ethnic groups. It hosts local government offices, including those of the chief and other local administrators. Permission to conduct activities in the community is always obtained through these offices."**

**Reviewer #3**

**Line 192**: The authors previously said in the section of method that S&C took 2 days and was conducted by six youths; Please can you clarify this?

The number of young people who conducted S&C in this intervention site must be specified; this must be clear for the 2 intervention sites

**Response**

**This was clarified. At the R. Naam the intervention site was small. Therefore, the clearing took three days while along the R. Yei where the intervention site was rather large it took 7 days. This has been revised in the text.**

**Reviewer #3**

**Line 189-190**: S&C performed 700 m upstream and 200 m downstream of the intervention sites. Does this mean that the section between upstream and downstream was not affected by this intervention?

**Response**

**The performance of S&C usually starts from the central point and extend to both downstream and upstream covering the central point. The intervention ensures that no section in the intervention site is left. The intervention therefore covered all the section between down and upstream.**

**Reviewer #3**

**Lines 240 et 244**: The authors should present the number of larvae collected per area of substrate

What is the meaning of ++++ in Table 1? The term “larvae and pupae” appears several times in the legend, and the authors need a clear way of presenting it.

**Response**

**++++ was an error should be +++, and this is a standard semi-quantitative way of expressing the number of larvae on a trailing vegetation or substrate whereby +++, means >50 larvae. We also revised the legend by deleting the term “larvae and pupae” from most of the areas and only left one at the beginning to guide readers. Under adult fly in the legend a revision was also made.**

**Reviewer #3**

**Lines 253-255**: The authors should move these sentences in the section of methods

**Response**

**Sentences were removed from the result section**

**We now write in the methods “Volunteers residing near the Naam and Yei Rivers were selected based on recommendations from local chiefs. The research team trained these individuals in basic blackfly collection and preservation techniques [8], and all were treated with ivermectin. A total of 14 vector collectors were trained: eight were deployed across four catching sites along the Yei River, and six were assigned to three sites along the Naam River. At each site, two collectors operated within 500 meters of known blackfly breeding sites.”**

**Reviewer #3**

**Line 256**: After giving the unit of the monthly biting rate or daily biting rate on the method section (bite per person per month or bite per person per day), use their abbreviations bi/p/m or bi/p/d in the section of results

**Response**

**We now mention the unit of the monthly biting rate or daily biting rate in the method section (****bite per person per month or bite per person per day), and use their abbreviations bi/p/m or bi/p/d in the section of results**

**Reviewer #3**

**Line 260** : Write per day

In table 2, Daily Biting Rate (DBR); replace DBR by (bi/p/d)

**Response**

**Done. DBR related to Table 2 under section “Collection of adult biting *Simulium*’ was revised to BI/P/D in all sentences where they appear in this section.**

**Reviewer #3**

**Lines 269-270**: The authors must move this sentence in the section of discussion

**Response**

**Sentence was deleted under results and moved to the discussion section**

**Reviewer #3**

**Lines 276-280**: That doesn't seem to convince me. There are already 2 control sites and one intervention site shown in the figure. Which of these are affected by the reduction you refer to in the 4 months following S&C? It seems rather contrary to me to take a good look at the figure and propose an appropriate comment.

**Response**

**We do agree with your observation. After critically reviewing Figure 4, we noted the reduction for three consecutive months was seen in Main bridge control site, whereas when assessing the impact using the Domilara control site, this reduction was only for two months. This revision was made in the text.**

**Reviewer #3**

On **line 192**, the authors say that S&C was implemented in March and on figure 6 the arrow is pointing to April. Can you clarify this misunderstanding? My comment about reduction rate of biting in Naam, comes back here

**Response**

**This was an error in the graphics, and has been corrected in Figure 6.**

**Reviewer #3**

Table 2 Write “degrees”

**Response**

**Degrees has been added besides Latitude and Longitude as unit of measurement in Table 1.**

**Reviewer #3**

**Lines: 335-338**: What makes you conclude that it's S. damnosum when you're talking about limited data? What makes you conclude that it's S. damnosum when you're talking about limited data?

**Response**

**The larva collected from different sites and examined both by hand lens and dissecting microscope was characterized by pronounced tubercles and spatulate scales. Using the identification keys provided in the WHO Manual (2023) it indicated that the species was *S.* *damnosum s.l*. Furthermore, gill filaments where dissected, and a banana shaped forms of filament were observed. These were the basis for our conclusion.**

**Reviewer #3**

**Line 371**: In this part of the discussion, the authors should mention the contamination of the intervention sites by the control sites, which are all nearby, as black flies can move over long distances

**Response**

**This suggestion was incorporated in the relevant section of the discussion.**

**We now state “The contamination of intervention sites by control sites in this study was attributed to the short distances between control and intervention sites. In Uganda, a country neighbouring South Sudan, *S. damnosum* s.l was reported to disperse up to a distance of 55 km from the breeding site [20].”**

**Reviewer #3**

**Line 440-441**: The authors should revise the sentence

**Response**

**This sentence was revised in the text.**

**We now write “However, suspected *S. damnosum* breeding on rocks complicated the intervention efforts”**

**Reviewer #3**

The authors need to review the indicators used to calculate the reduction rate (% exchange rate)

**Response**

**Our indicators for measuring the impact of S&C in this study included calculating the percentage change in MBR between baseline and in the month immediately following S&C using the formula: % change = [ (post intervention MBR – Baseline MBR) / Baseline MBR ] ×100. Additionally, we used the Mann-Kendall Trend test to capture the impact of S&C over several months. We find these two approaches appropriate.**

**Reviewer #4**

**Overall feedback**

This is a great work especially as slash and clear has not been overly exploited as a vector control tool. However, I had a bit of concern especially with the study design. It was unclear to me if this was a case control study or an intervention study (before-after).

**Response**

**The study was initiated as a clinical trial with intervention and control sites. However as the control sites were located close to the intervention sites a before-after analyses was considered as the preferred way to analyse the study.**

**Reviewer #4**

Secondly, in the abstract, I think the results and conclusion do not emphasize the purpose of the work. From my understand, the study was to assess the impact of the community-based vector control S&C. The other results such as the BR being higher in one season than the other, etc., are more secondary outcomes but they overshadowed the rationale behind the study. I think the main objective should stand out esp. in the abstract. This was however elaborated in the author summary.

**Response**

**We now state in the abstract “Monthly biting rates (MBR) were monitored during 8-14 months. Biting rates were compared between intervention and control sites between baseline and end of the study period.”**

**Reviewer #4**

The objectives of the study are not clearly articulated with a clear testable hypothesis.
A comparison is made between the baseline Monthly Biting Rate and the subsequent MBR for 14 months. It is difficult to imagine that this Slash and Clear could have an impact on the subsequent MBR during 14 months.

**Response**

**Our hypotheses, based on our experience with Slash and Clear in Maridi where there was only one breeding site at the Maridi Dam, was indeed that Slash and Clear could have an impact on the subsequent MBR during 14 months. However the study showed that this was not the case in Mvolo and Mundri.**

**Reviewer #4**

**Line review:**

Line 96: Double Sudan. Delete one

**Response**

**Done**

**Reviewer #4**

Line 71, 149, 153, etc.: Blackfly or Black fly? Could you select one and be consistent with that.

**Response**

**We now use only blackfly**

**Reviewer #4**

Line 159-161: Add that larval density at the breeding sites was determined, as you have indicated the infestation levels in the results (line 240; 243; 244…)

**Response**

**We incorporated the sentence in the text to read: “Larval density at the breeding sites was determined semi-quantitatively (<10 larva/pupae, 11-50 larvae/pupa, >50 larvae/pupae)”.**

**Reviewer #4**

Line168-169: How many collection points for each river? Will be good to mention.

**Response**

**The collection points were indicated for each River. Three collection points along R. Naam and four collection points along the Yei River.**

**Reviewer #4**

Line 191-192: Was the S&C for the 7 days done consecutively? If so, kindly indicate.

**Response**

**This was revised in the text to indicate that the S&C was done during 7 consecutive days.**

**Reviewer #4**

Line 302: Add GAM, which is then used in line 308.

**Response**

**We added GAM**

**Reviewer #4**

Line 308: The GAM model…, can you take out the “model”?

**Response**

**We removed model**

**Reviewer #5**: This paper by Thomson Luroni Lakwo et al. is a contribution of the experimentation of the impact of the Slash and Clear strategy on the control and elimination of onchocerciasis. This strategy has shown a significant reduction in the Simulium Monthly Biting Rate where it was implemented. In this study by Thomson Luroni Lakwo et al, a unique round of Slash and clear was conducted and the impact evaluated throughout the following 14 months, with the evaluation of the percentage change relative to baseline Monthly Biting Rates (MBR). Considering the cycle of Simulium damnosum around a month and the cycle of the vegetation along the fast-flowing rivers that grows after one month or so, one wonders what the hypothesis was, in hoping the one round Slash and clear could have a significant impact on Simulium daily or monthly biting rate throughout the following 14 months. Whatever the analyses are, or the model, it is difficult to conceive that one round of Slash and clear could have impact on Simulium Monthly Biting Rate and hence an impact on onchocerciasis transmission.

The results of this study are trouble message concerning this environmentally friendly vector control strategy. Even if the reduction of the biting rate in some experiments is modest, it remains that this strategy induced in most cases a significant reduction of the biting rates, with monthly or two monthly Slash and clear. Nobody can imagine that one round of Slash and Clear can impact either the biting rate or the onchocerciasis transmission.

**Response**

**In a previous study in Maridi (South Sudan), we documented that one round of S&C maintained monthly biting rates at significantly lower levels after several months because the sole blackfly breeding site had been destroyed (Ref: Jada SR, et al. Lancet Glob Health. 2023;11: e1260–e1268. doi:10.1016/S2214-109X(23)00248-6). We made assumptions that similar results could be obtained in Mundri West and Mvolo, but this was not the case since there were several breeding sites along the target rivers. Indeed, S&C intervention must be repeated regularly to obtain a significant reduction in biting rates in such settings.**

**Journal requirements**

1) Please ensure that the CRediT author contributions listed for every co-author are completed accurately and in full.

**Author contributions**

**Conceptualization: RC, SRJ, TLL. Data curation: TLL, SRJ, Field investigation: SRJ, TLL, PA, MT, CD, DGD. Methodology TLL, JSF. Supervision: TLL, RC. Software JSF. Funding acquisition: SRJ and RC. Project Administration: SRJ, Visualization: TLL, JSF. Formal analysis: TLL, JSF, MS, Validation: JSF, TLL, Writing original draft: RC, TLL, Writing – Review & Editing: RC, SRJ, TLL, JSF.**

2) Please upload all main figures as separate Figure files in .tif or .eps format.

**Done**

3) We have noticed that you have uploaded Supporting Information files, but you have not included a list of legends. Please add a full list of legends for your Supporting Information files after the references list.

**We now included a List of legends of supporting information files**

**Supplementary Material 1a (S1a):**

**Results of un-paired t-test statistic between intervention and control sites along Naam River in Mvolo County, Western Equatoria, South Sudan**

**Supplementary Material 1b. (S1b.):**

**Results of un-paired t-test statistic between intervention and control sites along Yei River in Mundri West County Western Equatoria, South Sudan.**

4) Some material included in your submission may be copyrighted.

**Nothing of the material is copyrighted.**

- Please confirm (a) that you are the photographer of Figure 5

**The co-author S Jada is the photographer of Figure 5**

- Figures 1 and 2. Please (a) provide a direct link to the base layer of the map

**The South Sudan shape files for Figures 1 and 2 was from a site called “****The Humanitarian Data Exchange”,** [**https://data.humdata.org/**](https://data.humdata.org/)

**This is now mentioned in the paper**

5) We note that your Data Availability Statement is currently as follows: "The data reported in the submitted manuscript are provided as part of the submitted article".

**We confirm that all raw data are available in the paper**

6) Please amend your detailed Financial Disclosure statement.

# **Funding source**

# **This work was supported by an R2HC grant to SRJ, Amref Health Africa, South Sudan (Project ID: 78719).The funders had no role in study design, data collection and analysis, decision to publish, or preparation of the manuscript.**

# **Competing interest**

**The authors have declared that no competing interests exist.**
