# Supplementary material for: A community-based vector control intervention “Slash and Clear” implemented in two onchocerciasis-endemic foci in South Sudan
Source: PLoS Negl Trop Dis. 2025 Jul 23;19(7):e0013309. doi: 10.1371/journal.pntd.0013309 (PMC12306731; doi:10.1371/journal.pntd.0013309)
Supplement: Attachment — Submitted filename: Feedback_S_C 2nd review.docx [file pntd.0013309.s006.docx]

A community-based vector control intervention “Slash and Clear” implemented in two

onchocerciasis-endemic foci in South Sudan

PNTD-D-25-00522

I am satisfied with the responses from the author. I have no further recommendations to made.

Many thanks
